# Vibroacoustic signatures: proof of concept for simple material characterization during needle interventions

**DOI:** 10.1007/s11548-025-03492-0

**Published:** 2025-08-04

**Authors:** K. Steeg, W. Serwatka, D. Rzepka, H. Oran, O. B. Özdil, K. Heryan, G. A. Krombach, M. H. Friebe

**Affiliations:** 1 Department of Diagnostic and Interventional Radiology, University Hospital Giessen, Justus-Liebig-University Giessen, Klinikstraße 33, 35392 Giessen, Germany; 2 Faculty of Computer Science, AGH University Kraków, 30-059 Kraków, Poland; 3 INKA Innovation Lab, Faculty of Medicine, Otto-Von-Guericke-University, Magdeburg, Germany

**Keywords:** Vibroacoustic sensing, Minimal invasive procedures, Tissue differentiation, Tissue classification

## Abstract

**Purpose:**

This proof of concept investigates the potential of vibroacoustic signals, originating from a needle tip during puncturing, as a method to differentiate ex vitro materials based on their structural characteristics. The main research question is whether the number and distribution of amplitude events in vibroacoustic waveforms correlates with the material structure, offering a feasible approach for a future real-time tissue differentiation in minimally invasive procedures.

**Methods:**

Two types of synthetic foams with different air pocket densities were punctured using a standard Quincke lumbar needle with cutting bevel. Vibroacoustic signals were recorded during the puncture, and the number of amplitude events detected per unit distance was analyzed. The structural differences of the foams were quantified by counting the number of air pockets per unit length. Part of the study was to also consider the impact of puncture / insertion speed on the signal characteristics.

**Results:**

A significant correlation was observed between the air pocket density of the foams and the number of detected events per unit distance. The foam with a higher air pocket density produced more detected events compared to the one with a lower density. Insertion speed of the needle did not significantly impact the number of detected events.

**Conclusion:**

The findings demonstrate that vibroacoustic signals hold information that allows the differentiation of materials based on their structural properties, laying the foundation for further research into their application in real-time tissue differentiation. Integrating vibroacoustic sensing into minimally invasive procedures could provide valuable additional information about tissue composition and integrity, potentially improving surgical precision in procedures such as tumor biopsies. Further research is needed to validate these findings with biological tissues and refine the technology for clinical use.

## Introduction

During minimally invasive needle-based interventions, understanding tissue properties is critical when navigating to the target sites [1]. Particularly in interventions like biopsies or tumor resections, obtaining high-quality samples is critical for diagnosis and treatment planning, yet it is often compromised because most carcinomas are a heterogeneous mixture of cells, making it challenging to assess tissue characteristics in real time [2]. In traditional open surgeries, surgeons obtained tissue property information through direct view of the anatomic landscape and tactile feedback from palpation, which allowed them to adjust the procedure based on variations in texture, resistance and elasticity [1, 3]. In contrast, in minimally invasive procedures performed through small incisions, information from these sensory channels is restricted [1, 3, 4]. Although imaging modalities such as ultrasound (US), fluoroscopy, and magnetic resonance imaging (MRI) offer real-time guidance, they are limited by resolution, availability, cost, radiation exposure and fail to provide real-time feedback on tissue integrity [5–8].

Several techniques exist that aim to compensate for the loss of tactile feedback in minimally invasive procedures. Some approaches embed optical fibers into the needle to analyze tissue density and scattering properties 1–2 mm ahead of the tip, enabling tissue classification or high-resolution tissue imaging [2, 9]. However, these systems require specialized needles, making the procedures less cost-effective and flexible. Another method integrates laser beams into US probes for photoacoustic sensing without altering the needle [10, 11]. While this improves penetration depth, it comes with the cost of reduced resolution and susceptibility to air bubbles [11].

To address these limitations, we propose to use vibroacoustic signals, a sensing approach inspired by human tactile perception, as a complementary modality for real-time assessment of material properties [12, 13]. In humans, tactile texture perception incorporates two subsenses of the sense of touch: the spatial sense, which allows for the discrimination of coarse textures and the vibration sense, which enables the tactile characterization of fine textures [14]. Fine textures are sensed through friction-induced vibration (FIV) created by the dynamic movement of the finger across a surface [15]. These high-frequency skin vibrations activate mechanoreceptors, particularly Pacinian afferents, producing precise temporal spiking patterns that encode surface features [16–18]. Although these patterns dilate or contract with decreased or increased scanning speed, they preserve material identity, enabling speed-invariant perception of fine textures [15, 16, 19]**.**

Based on this principle, it is hypothesized that mechanical interactions between a needle and a material generate vibroacoustic signals similar to FIV, offering a promising approach for extracting information about material structure inspired by the human sense of vibration [12, 13]. This hypothesis is supported by the previous studies that explored acoustic sensing to characterize puncture dynamics, tissue–tool interactions, and tissue classification [13, 20–22]. For example, vibroacoustic signals produced during surface palpation with a laparoscope were used to differentiate between tissues such as bone, tendon, and liver [13]. However, in that setup, the instrument was free to oscillate and not fixed along the shaft. In contrast, tools used in minimally invasive procedures are often partially or fully constrained by surrounding tissue, which attenuate and modify their resonating behavior compared to palpation. Another study used vibroacoustic signals from manually punctured phantoms to train AI models for tissue classification, demonstrating that these signals encode tissue-specific information [22]. However, the signals require a more detailed analysis to identify interpretable features for standardized tissue classification.

Because temporal vibration patterns resemble the sensory processing of fine textures, we propose that analyzing the number and distribution of amplitude peaks in vibroacoustic signals provides a method for simple material characterization. In this study, signals are captured directly from the needle during puncture and analyzed to infer material characteristics such as density and structural composition.

The central hypothesis of this novel diagnostic technique is that each material possesses a unique vibroacoustic signature that holds information about structural features and enables event-based material differentiation, even across varying puncture speeds. This study provides proof of concept that vibroacoustic signals captured directly from the needle are a viable modality for simple structural and event-based material differentiation during needle interventions using synthetic foams with varying properties.

## Materials and methods

### Clip-on prototype device and experimental setup

The experimental setup is based on puncturing foams with different structural properties, represented by varying air pocket densities. The hypothesis is that each air pocket generates an amplitude event (peak) upon being crossed, and the number of peaks per traveled unit distance correlates with the structure of the punctured material.

**Fig. 1:**
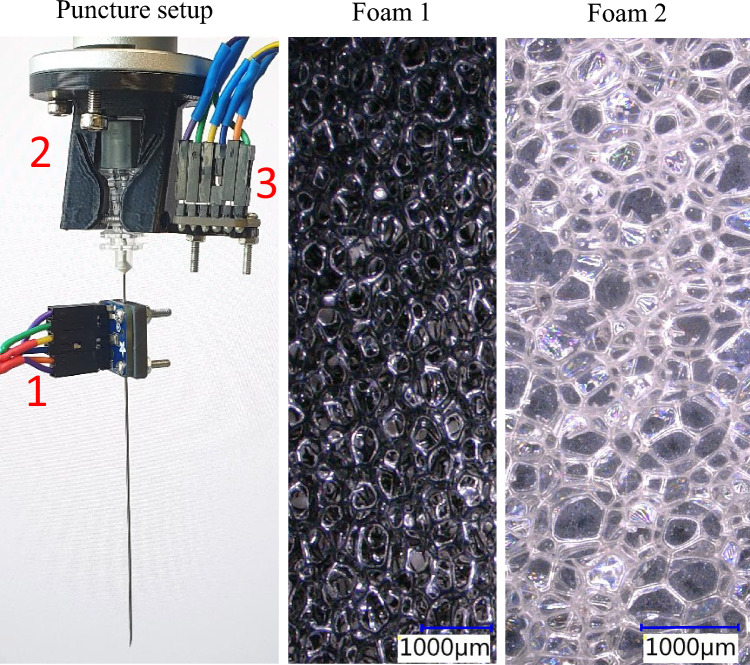
Experimental setup for needle punctures along microscopic images of both foam structures. A 22G Quincke needle equipped with a sensor device via a 3D-printed clip-on (1) was inserted into the foams using a robotic arm with a 3D-printed needle holder (2). An A4 sheet of paper was used to mark the needle entry points into the foams. Vibroacoustic signals were recorded in parallel with video recordings and an external microphone recorded ambient noise (3). Both foams were viewed at up to 10 × magnification. Foam 1 shows smaller and more air pockets per 1000 µm compared to Foam 2

A custom-developed clip-on system was utilized throughout the study to capture vibroacoustic signals during needle puncture. The system integrates two MEMS microphones (Adafruit I2S MEMS Microphone Breakout, SPH0645LM4H), selected for its small form factor, low cost, and compatibility with Raspberry Pi for data acquisition. A three-dimensional (3D) printed clip-on holder was designed to attach the sensors to the proximal end of medical tools, such as needles, without altering the needle itself. The needle shaft is positioned directly in front of the sound hole of one microphone and held in place by two small screws (Fig. 1 left). This physical contact allows utilizing the needle as a resonator that transfers vibroacoustic signals generated at the needle tip to the microphone’s membrane. The second microphone is only attached to the holder but not coupled to the needle itself, allowing for distinguishing actual vibroacoustic signals from acoustic noise generated during the puncture and external noise.

Puncture experiments were conducted using a Franka Emika robot arm to ensure precise and repeatable movements. A 22G spinal needle with a Quincke tip was held by the robot at its standardized luer lock mechanism and performed insertions into a foam phantom at a 90° angle. The phantom consisted of two types of foam materials stacked on top of each other and separated by a standard A4 sized paper that was anticipated to provide a distinct high amplitude signal upon penetration. On top of the first foam, another A4 sized paper was placed. Each foam was 15 mm thick, and the phantom was punctured 70 times at the two controlled speeds of 10 mm/s and 20 mm/s, to evaluate the influence of puncture velocity on the vibroacoustic signals produced during needle insertion. These speeds resemble medium and higher-end insertion velocities during clinical procedures by still providing a noticeable difference [23].

The foams were chosen based on their structural differences, specifically the concentration of air pockets. These differences were visually assessed under 10 × microscopy, providing a basis for correlating material structure with the vibroacoustic signals captured during needle penetration. A single observer counted the number of air pockets per 10 mm along 20 randomly chosen paths in both foams.

### Vibroacoustic signal recording

The clip-on system’s MEMS microphone captured real-time vibroacoustic signals as RIFF WAVE (.wav) files with sample rate 48 kHz controlled from a custom-made laboratory application during the robot-assisted needle insertions. Additionally, a video camera recorded the punctures in parallel to allow for signal annotations later. Signal waveforms were processed for each foam individually, and the number of peaks per unit distance was computed to evaluate whether they corresponded to foam air pocket penetrations and material interactions. To remove recording artifacts and to eliminate a slow-varying offset introduced by the analog acquisition system, a highpass DC blocking filter set to 75 Hz was applied.

### Signal separation

A custom-made laboratory application was used to synchronize video and audio recordings from experiments based on an external trigger present in both recordings. After synchronization, video recordings were annotated manually to serve as ground truth for punctures in the audio signal.

Initial analysis of the puncture period revealed two distinct regions of increased peak density within each recording, corresponding to the needle’s entry into the two foams. In these intervals, the needle contacted the A4 paper on top of the foam without immediate perforation. Instead, the paper and the foam were compressed until the applied force exceeded the resistance and the needle penetrated paper and foam. At this point, the compression of the foam rapidly relaxed, resulting in the needle piercing multiple air pockets in a short time span that generated a high-density burst of peaks.

Given the hypothesis that each foam would produce a unique vibroacoustic signature characterized by the distribution of peaks per unit distance, the recorded waveform was divided into two segments corresponding to each foam. The segmentation was achieved by splitting the waveform into windows of equal duration, each referring to 1 mm of traveled distance, calibrated according to the needle's speed (in mm/s). Peaks within these windows were detected based on a minimum amplitude threshold to distinguish relevant peaks from background noise. A small amplitude threshold of 0.006 was set based on experimental observation. This value was chosen because it yielded the clearest separation between the two distinct peaks in the peaks/mm plot, corresponding to the entry into each foam. Background noise was not measured explicitly, but no consistent noise floor was observed that would justify a different fixed cutoff. No additional filter was applied during this step, as the peaks were temporally close together and further smoothing risked suppressing relevant signal features.

Corresponding to intervals with high-density of peaks, two maxima in the number of peaks/mm marked the entry points of the needle into the foams. Based on the time points of these maxima, the waveforms of the foams were separated by calculating the approximate start and end sample indices of each foam. This approach ensured separation of the signals, allowing for individual analysis of each foam's vibroacoustic characteristics.

### Audio signature analysis

Both separated waveforms were analyzed similarly to be able to compare peak counts/10 mm between foams. An averaging filter was used to compute the amplitude envelope of each foam signal to smooth out extrema close to each other and minimize the influence of small amplitude fluctuations caused by environmental noise. The best numerator coefficient vector for the averaging filter was found by computing the Receiver Operating Characteristic Area Under the Curve (ROC AUC) score from values 22 to 75 in steps of 5 for each speed setting. Subsequently, the number of peaks/10 mm was calculated with the threshold automatically adapted to the mean value of the envelope. The adaptive threshold ensured that only peaks significantly higher than the basic noise level were counted as events. At the beginning of each signal, an accumulation of peaks occurred, which helped to find the beginning of the foam puncture but was excluded from the peak counting because the peak density in this area was not representative for the rest of the signals.

## Results

This study aimed to quantify the relationship between vibroacoustic signal events and the structural characteristics of foam materials during needle puncture. The analysis focused on the mean event counts per unit distance, alongside their standard deviations, across two puncture speeds.

**Fig. 2:**
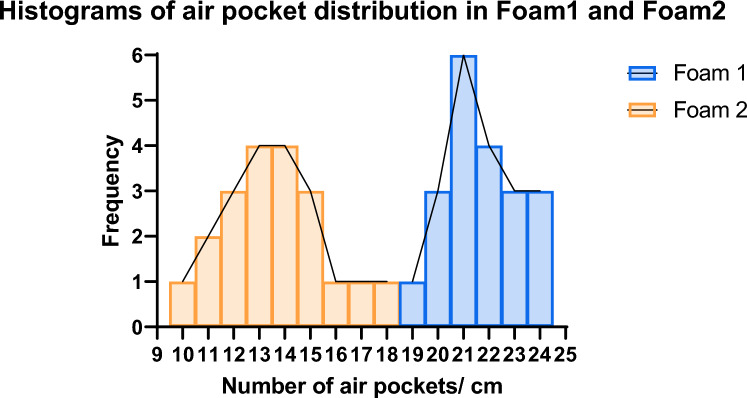
Distribution of air pockets in Foam 1 and Foam 2. Air pockets were counted along 20 randomly chosen paths through both foams (10 paths at each of two positions)

The structural characteristics of the two foams were visually assessed by counting air pockets/10 mm along 20 randomly chosen paths through the foams. Among locations, the air pocket counts remained similar but yielded significant differences between the foams (Fig. 2). Foam 1 exhibited an average of 21.7 ± 1.455 air pockets/10 mm, equating to approximately one air pocket every 0.46 mm. In contrast, Foam 2 showed a lower average of 13.60 ± 2.037 air pockets/10 mm, or one air pocket every 0.73 mm.

**Fig. 3:**
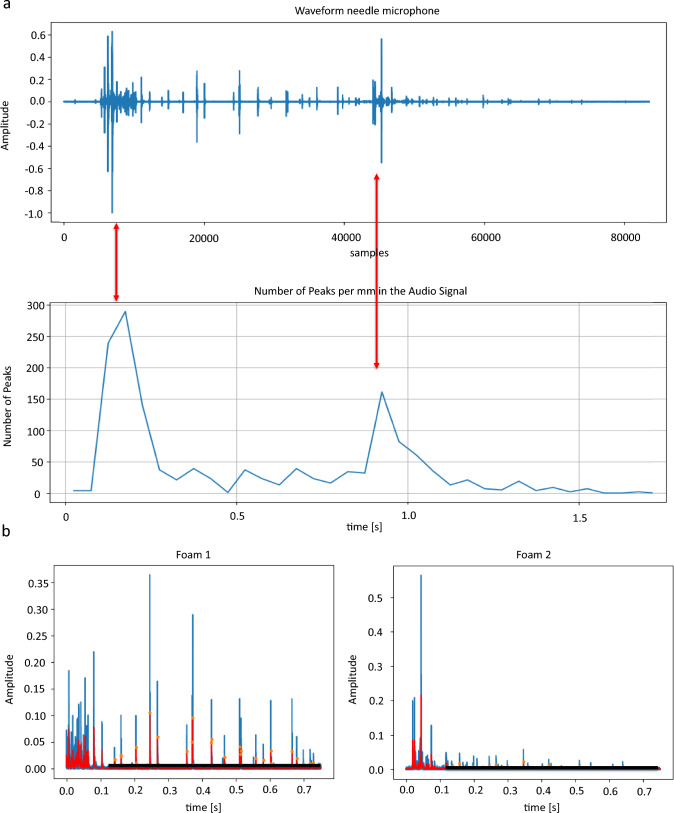
Raw waveform and number of peaks per mm of one audio signal for 10 mm/s. Two maxima marked the penetration of the A4 paper (red arrows) (**a**). Foams were separated based on the highest number of peaks per mm. Waveforms after the averaging filter (red) and identified and counted peaks (orange dots). The height is indicated as a horizontal black line (**b**)

The interval of foam puncture was identified according to manual annotations based on video files. Once extracted, the number of peaks/mm in the puncture period was calculated (Fig. 3A). The number of peaks/mm was then calculated, revealing two distinct maxima corresponding to the needle's entry points into the foams. Based on these maxima, the foam signals were separated (Fig. 3B). To avoid bias from the initial accumulation of peaks at the puncture's entry point, this segment was excluded from the analysis because the peak density in this area did not represent the rest of the signals. Therefore, a total needle travel distance between 11 and 12 mm was analyzed for peak events instead of the full 15 mm. To minimize noise and enhance peak detection accuracy, an averaging filter was applied to the vibroacoustic signals (Fig. 3C). To evaluate the optimal filter for differentiating between foams, filter performance was assessed through peak count analysis and classification metrics.

**Fig. 4:**
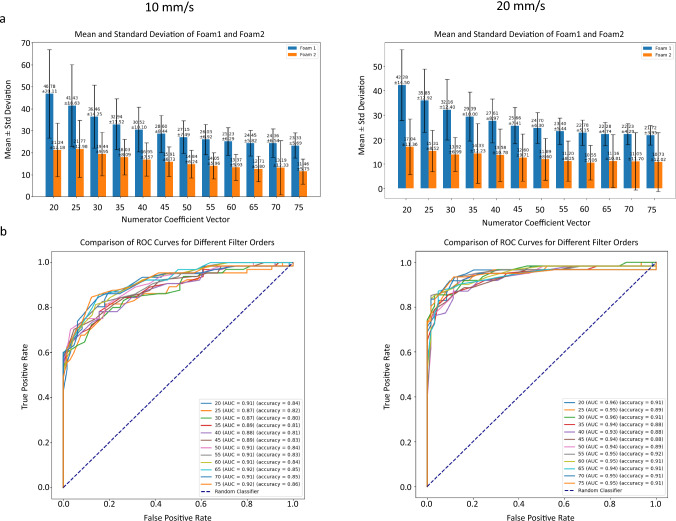
Mean and standard deviation of two foams with different filter orders (**a**) alongside their AUC-ROC for different filter orders and accuracy values (**b**)

Twelve filter orders, ranging from 20 to 75 in 5-unit increments, were tested and their corresponding AUC-ROC curves and accuracy were computed to identify the optimal filter for distinguishing the two foams (Fig. 4). For Foam 1, peak counts decreased by approximately half with increasing filter order, stabilizing at around 23 peaks per unit distance for both speed settings at filter order 60 (Fig. 4A). Foam 2 exhibited a similar trend, with peak counts plateauing at around 12 peaks per unit distance at filter order 60, regardless of speed. The relation of peak counts between the two foams remained consistent, with Foam 2 showing half the number of peaks compared to Foam 1.

Since the relation of peak counts between foams remained consistent across filter orders and speeds, AUC-ROC curves and accuracy values showed minimal variation (Fig. 4B). Filter order 65 was selected for its optimal balance of signal smoothing and peak preservation, closely matching the air pocket distribution observed in microscopy images (see Fig. 2). Event counts for this filter order were analyzed to characterize each foam's vibroacoustic signature.

**Fig. 5:**
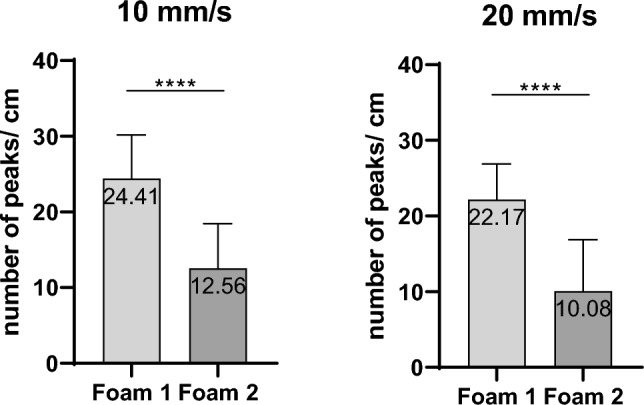
Vibroacoustic Event Counts at filter order 65 (closest to actual numbers)

At filter order 65, event counts were calculated per 10 mm of needle travel for both foams and outliers were excluded (Fig. 5). Foam 1 generated an average of 24.4085 ± 5.783 events/10 mm at 10 mm/s and 22.1726 ± 4.703 events/10 mm at 20 mm/s. Foam 2 produced fewer events, with averages of 12.5648 ± 5.874 events/10 mm at 10 mm/s and 10.08 ± 6.792 events/10 mm at 20 mm/s. The relation between Foam 1 and Foam 2 remained significant across the speeds of 10 mm/s and 20 mm/s, indicating that those two puncture speeds did not affect the number of events per unit distance.

## Discussion

This proof-of-concept correlated events per unit distance with the material structure. By puncturing foams with varying air pocket concentrations and analyzing the amplitude events in the resulting vibroacoustic waveforms, it was shown that the distribution of these events reflect key structural features of the material, offering a potential new method for real-time event-based tissue differentiation.

In this study, the temporal peak distribution of vibroacoustic signals was analyzed by examining the number of peaks per traveled unit distance. The analysis of foam structure revealed a correlation between the number of air pockets/10 mm and the number of amplitude peaks detected in the signal. Specifically, Foam 1, which had an average of 21.7 air pockets/10 mm, produced a significantly higher number of peaks/10 mm compared to Foam 2, which had an average of 13.6 air pockets/10 mm. This correlation was consistently observed through both visual counting and signal analysis, underscoring the significant impact of material structure on the vibroacoustic response. These findings show that temporal peak patterns provide valuable insights into material properties akin to how the human sense of touch interprets fine textures.

Consequently, the influence of different puncture speeds on the vibroacoustic signal was investigated. A robot arm was used to puncture a foam phantom at two constant speeds: 10 mm/s and 20 mm/s. The results showed that faster puncture speeds produced more peaks within a shorter time frame, which was expected, as the needle punctured the air pockets more quickly. This was especially observed at the beginning of each puncture, where the compression and rapid relaxation of the foam resulted in an interval with a higher density of peaks. At this point, the needle passed the air pockets faster than with the average speed setting but with unknown exact speed and therefore, this interval was removed for further analysis. Similarly, other studies observed an increased root-mean-square velocity (RMS velocity) with higher movement speeds [18]. The RMS velocity represents the energy of a system that is related to the frequency and amplitude of vibrations [18]. Therefore, a higher number of distinct peaks per distance also contributes to a higher RMS velocity. When the number of peaks was calculated relative to the distance traveled rather than the time, the puncture speed did not significantly alter the number of events per unit distance. This supports the hypothesis that the events are directly related to the number of air pockets encountered and thus correspond to the material structure. The stability of these results across varying speeds highlights the reliability of vibroacoustic signals for real-time applications, provided the movement speed is known.

Apart from different puncturing speeds, another important variable in clinical settings is the type of needle used, as its bevel shape can influence the needle's interaction with the material and thus FIV. The Quincke style needles used in this study are frequently used for spinal anesthesia and lumbar punctures featuring a sharp cutting bevel that facilitates easy penetration through skin, tissue, and ligaments. Therefore, those conventional needles are often referred to as “cutting needles” [24]. However, Quincke needles are known to often cause postdural puncture headache (PDPH) by creating tension on the meninges aroused by the hole created in the dural tissues [25]. An alternative is offered by so-called non-cutting needles with a blunt pencil point tip, such as the Sprotte or Whitacre models. Those atraumatic needles are designed to spread tissues rather than cut them, therefore significantly reducing the risk of PDPH [24, 25]. Since vibroacoustic signals arise from friction between the needle and tissue, it is hypothesized that a different needle tip, especially when changing from a cutting to a non-cutting model, could alter the signal profile, specifically reducing the number of peaks/10 mm. Even in synthetic foams, non-cutting needles are likely to displace rather than puncture fibers and air pockets, resulting in fewer signal events. This hypothesis merits further experimental validation.

While this study demonstrated the feasibility of using information from vibroacoustic signals to differentiate materials, several limitations that must be addressed to fully realize the potential of this approach. The experiments were confined to synthetic foam materials, limiting the generalizability of the findings. While the proof of concept demonstrated the viability of peak number and distribution as suitable audio features for foam differentiation, broader validation across a wider range of materials, especially biological tissues, is essential. Most tissues are a heterogeneous composition of different cell types, and therefore, it remains to be investigated which structural or cellular features are most suitable for biological tissue characterization.

In this study, punctures were performed at two constant speeds (10 and 20 mm/s) using a 22G Quincke needle at a 90° angle with a robotic puncture setup. This standardization ensured reproducibility but does not reflect clinical variability in puncture angle, speed, and needle type. To address this limitation, we are developing a video-based method to analyze the speed of robotic and manual needle insertions using calibration markers retrospectively. However, real-time application requires further development. Although only a single needle type was used, the clip-on sensor is compatible with a wide range of needles, including larger diameters. While resonance characteristics may vary across needle types, core material features should remain constant. The analyzed materials were only 15 mm thick, but in clinical scenarios, relevant structures often lie at greater depths (e.g., the peritoneum). Although signal intensity may be attenuated with increasing puncture depth, it is hypothesized that characteristic features remain. This is supported by previous work that successfully recorded vibroacoustic data from a 150 mm Veress needle and biopsy needles [20]. Future work should examine these factors systematically and define detection limits under realistic conditions.

In practice, the ability to detect subtle material transitions can support clinicians in tasks such as optimizing the taking of good biopsy samples, particularly in situations where margins between healthy and malignant tissue are poorly defined. The core idea of this current approach is to detect those transitions between structurally distinct tissues by finding changes in peak patterns. Another potential application is the more accurate navigation and placement of the needle in challenging interventions. For example, in peritoneum puncture, it is not clearly indicated when the target site is reached. By augmenting and interpreting the vibroacoustic signal that is created when entering the peritoneal cavity, the safety and speed of the procedure can be enhanced [20]. Furthermore, real-time feedback from vibroacoustic signals could assist clinicians in adjusting needle trajectory, avoiding non-target areas, or confirming that critical zones have been reached. To work toward this integration, future research should build on this proof of concept and extend the investigations on vibroacoustic properties to more diverse materials, primarily focusing on biological tissues such as fat, muscle, or tumors.

The foams in this study were not intended to directly replicate specific biological tissues, but rather to represent structural variation in a simplified and controlled environment, and to demonstrate how vibroacoustic peak patterns depend on material structure. The air-filled pockets in the foams serve as examples of microstructural features that are hypothesized to be found in biological tissues as well. For instance, the alveolar cavities of the lung are frequently described as foam-like structures, and, in the context of fluid-filled compartments, the alternating architecture of hepatocytes and sinusoids in the liver provides a comparable structural pattern [26, 27].

Similar to tissues, which are comprised of a heterogenous mixture of different cell types, vibroacoustic signatures are likely composed of multiple audio features summing up to a more complex audio signature. Due to that, determining and assessing the utility of peak number and distribution as one part of this signatures is a critical next step for adopting this technology into clinical practice. Identifying reliable and specific audio features is essential for comprehensively describing distinct material patterns. Additionally, exploring the effects of environmental factors, procedural variables, and needle variants on vibroacoustic responses will enhance the robustness of the technology. Developing standardized protocols for consistent signal detection across clinical conditions will pave the way for integrating vibroacoustic sensing into medical tools to enhance surgical precision through improved differentiation of tissue types.

In conclusion, the findings in this proof of concept demonstrate that vibroacoustic signals hold information to differentiate between materials based on their structural compositions. This proof of concept demonstrated a direct correlation between the frequency of detected amplitude events in vibroacoustic signals and the material structure, laying a foundation for further exploration into real-time event detection and tissue differentiation during minimally invasive procedures. Further research has the potential to refine the method and expand its application to more complex and clinically relevant materials.

## References

[1] Colan J, Davila A, Hasegawa Y (2022) A review on tactile displays for conventional laparoscopic surgery. Surgeries 3:334–346. 10.3390/surgeries3040036

[2] Hariri LP, Adams DC, Applegate MB et al (2019) Distinguishing tumor from associated fibrosis to increase diagnostic biopsy yield with polarization-sensitive optical coherence tomography. Clin Cancer Res 25:5242–5249. 10.1158/1078-0432.CCR-19-0566 31175092 10.1158/1078-0432.CCR-19-0566 PMC6726561

[3] Westebring – van der Putten EP, Goossens RHM, Jakimowicz JJ, Dankelman J (2008) Haptics in minimally invasive surgery – a review. Minimally Invasive Therapy & Allied Technologies 17:3–16. 10.1080/13645700701820242 10.1080/13645700701820242 18270873

[4] Karangelis D, Androutsopoulou V, Tzifa A et al (2021) Minimally invasive cardiac surgery: in the pursuit to treat more and hurt less. J Thorac Dis. 10.21037/jtd-21-1498 34992800 10.21037/jtd-21-1498 PMC8662468

[5] Maddali MM, Arora NR, Chatterjee N (2017) Ultrasound guided out-of-plane versus in-plane transpectoral left axillary vein cannulation. J Cardiothorac Vasc Anesth 31:1707–1712. 10.1053/j.jvca.2017.02.011 28416391 10.1053/j.jvca.2017.02.011

[6] Sung JM, Jun YE, Jung YD, Kim KN (2023) Comparison of an ultrasound-guided dynamic needle tip positioning technique and a long-axis in-plane technique for radial artery cannulation in older patients: a prospective, randomized, controlled study. J Cardiothorac Vasc Anesth 37:2475–2481. 10.1053/j.jvca.2023.08.138 37741770 10.1053/j.jvca.2023.08.138

[7] Takeshita J, Nishiyama K, Fukumoto A, Shime N (2019) Comparing combined short-axis and long-axis ultrasound-guided central venous catheterization with conventional short-axis out-of-plane approaches. J Cardiothorac Vasc Anesth 33:1029–1034. 10.1053/j.jvca.2018.08.005 30269888 10.1053/j.jvca.2018.08.005

[8] Wang D, Ma D, Wong ML, Wáng YXJ (2015) Recent advances in surgical planning & navigation for tumor biopsy and resection. Quant Imaging Med Surg 5:640–648. 10.3978/j.issn.2223-4292.2015.10.03 26682133 10.3978/j.issn.2223-4292.2015.10.03 PMC4671974

[9] Surazynski L, Hassinen V, Nieminen MT et al (2024) Real-time tissue classification using a novel optical needle probe for biopsy. Appl Spectrosc 78:477–485. 10.1177/00037028241230568 38373402 10.1177/00037028241230568 PMC11070118

[10] Wei C-W, Nguyen T-M, Xia J et al (2015) Real-time integrated photoacoustic and ultrasound (PAUS) imaging system to guide interventional procedures: Ex Vivo study. IEEE Trans Ultrason Ferroelectr Freq Control 62:319. 10.1109/TUFFC.2014.006728 25643081 10.1109/TUFFC.2014.006728 PMC4610852

[11] Yu Y, Feng T, Qiu H et al (2024) Simultaneous photoacoustic and ultrasound imaging: a review. Ultrasonics 139:107277. 10.1016/j.ultras.2024.107277 38460216 10.1016/j.ultras.2024.107277

[12] Illanes A, Schaufler A, Sühn T et al (2020) Surgical audio information as base for haptic feedback in robotic-assisted procedures. Curr Dir Biomed Eng. 10.1515/cdbme-2020-0036

[13] Sühn T, Esmaeili N, Spiller M et al (2023) Vibro-acoustic sensing of tissue-instrument-interactions allows a differentiation of biological tissue in computerised palpation. Comput Biol Med 164:107272. 10.1016/j.compbiomed.2023.107272 37515873 10.1016/j.compbiomed.2023.107272

[14] Katz D (2013) The world of touch. Psychology Press, New York

[15] Cesini I, Ndengue JD, Chatelet E et al (2018) Correlation between friction-induced vibrations and tactile perception during exploration tasks of isotropic and periodic textures. Tribol Int 120:330–339. 10.1016/j.triboint.2017.12.041

[16] Boundy-Singer ZM, Saal HP, Bensmaia SJ (2017) Speed invariance of tactile texture perception. J Neurophysiol 118:2371–2377. 10.1152/jn.00161.2017 28724777 10.1152/jn.00161.2017 PMC5646196

[17] Weber AI, Saal HP, Lieber JD et al (2013) Spatial and temporal codes mediate the tactile perception of natural textures. Proc Natl Acad Sci 110:17107–17112. 10.1073/pnas.1305509110 24082087 10.1073/pnas.1305509110 PMC3800989

[18] Long KH, Lieber JD, Bensmaia SJ (2022) Texture is encoded in precise temporal spiking patterns in primate somatosensory cortex. Nat Commun 13:1311. 10.1038/s41467-022-28873-w 35288570 10.1038/s41467-022-28873-w PMC8921276

[19] Greenspon CM, McLellan KR, Lieber JD, Bensmaia SJ (2020) Effect of scanning speed on texture-elicited vibrations. J R Soc Interface 17:20190892. 10.1098/rsif.2019.0892 32517632 10.1098/rsif.2019.0892 PMC7328380

[20] Spiller M, Esmaeili N, Sühn T et al (2024) Enhancing veress needle entry with proximal vibroacoustic sensing for automatic identification of peritoneum puncture. Diagnostics (Basel) 14:1698. 10.3390/diagnostics14151698 39125574 10.3390/diagnostics14151698 PMC11311580

[21] Ostler D, Seibold M, Fuchtmann J et al (2020) Acoustic signal analysis of instrument–tissue interaction for minimally invasive interventions. Int J CARS 15:771–779. 10.1007/s11548-020-02146-7 10.1007/s11548-020-02146-7 PMC7261275 32323212

[22] Heryan K, Serwatka W, Sorysz J, et al (2023) Tissue Classification Using Data from Vibroacoustic Signals Produced from Needle-Tissue Interaction. In: 2023 IEEE EMBS Special Topic Conference on Data Science and Engineering in Healthcare, Medicine and Biology. pp 185–186

[23] van Gerwen DJ, Dankelman J, van den Dobbelsteen JJ (2012) Needle–tissue interaction forces – a survey of experimental data. Med Eng Phys 34:665–680. 10.1016/j.medengphy.2012.04.007 22621782 10.1016/j.medengphy.2012.04.007

[24] Kirschner JS, Furman MB (2018) Chapter 2 - Needle Techniques. In: Furman MB (ed) Atlas of Image-Guided Spinal Procedures (Second Edition). Elsevier, pp 19–26

[25] Xu H, Liu Y, Song W et al (2017) Comparison of cutting and pencil-point spinal needle in spinal anesthesia regarding postdural puncture headache. Medicine (Baltimore) 96:e6527. 10.1097/MD.0000000000006527 28383416 10.1097/MD.0000000000006527 PMC5411200

[26] Panwar A, Das P, Tan LP (2021) 3D hepatic organoid-based advancements in liver tissue engineering. Bioengineering (Basel) 8:185. 10.3390/bioengineering8110185 34821751 10.3390/bioengineering8110185 PMC8615121

[27] Prange HD (2003) Laplace’s law and the alveolus: a misconception of anatomy and a misapplication of physics. Adv Physiol Educ 27:34–40. 10.1152/advan.00024.2002 12594072 10.1152/advan.00024.2002

